# Brazilian Maternal and Child Nutrition Consortium: establishment, data harmonization and basic characteristics

**DOI:** 10.1038/s41598-020-71612-8

**Published:** 2020-09-10

**Authors:** Thaís Rangel Bousquet Carrilho, Dayana Rodrigues Farias, Mônica Araújo Batalha, Nathalia Cristina Freitas Costa, Kathleen M. Rasmussen, Michael E. Reichenheim, Eric O. Ohuma, Jennifer A. Hutcheon, Gilberto Kac, Brazilian Maternal and Child Nutrition Consortium, Adauto Emmerich Oliveira, Ana Paula Esteves-Pereira, Ana Paula Sayuri Sato, Antônio Augusto Moura da Silva, Bárbara Miranda Ferreira Costa, Claudia Leite de Moraes, Claudia Saunders, Cristina Maria Garcia de Lima Parada, Daniela da Silva Rocha, Denise Petrucci Gigante, Edson Theodoro dos Santos-Neto, Elisa Maria de Aquino Lacerda, Elizabeth Fujimori, Fernanda Garanhani Surita, Isaac Suzart Gomes-Filho, Isabel Oliveira Bierhals, Jane de Carlos Santana Capelli, José Guilherme Cecatti, Juliana dos Santos Vaz, Juraci Almeida Cesar, Marco Fábio Mastroeni, Maria Antonieta de Barros Leite Carvalhaes, Mariângela Freitas da Silveira, Marlos Rodrigues Domingues, Mayra Pacheco Fernandes, Michele Drehmer, Mylena Maciel Gonzalez, Patrícia de Carvalho Padilha, Renato Passini Junior, Renato Teixeira Souza, Ronaldo Fernandes Santos Alves, Rosângela Fernandes Lucena Batista, Silmara Salete de Barros Silva Mastroeni, Silvia Regina Dias Medici Saldiva, Simone Seixas da Cruz, Sirlei Siani Morais, Sotero Serrate Mengue

**Affiliations:** 1 Nutritional Epidemiology Observatory, Josué de Castro Nutrition Institute, Federal University of Rio de Janeiro, Avenida Carlos Chagas Filho 373/CCS, bloco J, 2 andar, sala 29, Cidade Universitária, Ilha do Fundão, Rio de Janeiro, RJ 21941-902 Brazil; 2 Division of Nutritional Sciences, Cornell University, 227 Savage Hall, Ithaca, NY 14850 USA; 3 Department of Epidemiology, Institute of Social Medicine, Rio de Janeiro State University, Rua São Francisco Xavier, 524, 7 andar, Bloco D, Sala 7018, Maracanã, Rio de Janeiro, RJ 20550-013 Brazil; 4 Maternal, Adolescent, Reproductive and Child Health (MARCH) Centre, London School of Hygiene and Tropical Medicine, Keppel Street, London, WC1E 7HT UK; 5 Centre for Tropical Medicine and Global Health, Nuffield Department of Medicine, University of Oxford, Peter Medawar Building for Pathogen Research (PMB), South Parks Road, Oxford, OX1 3SY UK; 6 Department of Obstetrics and Gynaecology, Faculty of Medicine, University of British Columbia, Suite 930, 1125 Howe Street, Vancouver, BC V6Z 2K8 Canada; 7 Postgraduate Program in Collective Health, Federal University of Espírito Santo, Vitoria, ES Brazil; 8 Department of Epidemiology and Quantitative Methods in Health, Sérgio Arouca National School of Public Health, Oswaldo Cruz Foundation, Rio de Janeiro, RJ Brazil; 9 Department of Epidemiology, School of Public Health, University of São Paulo, São Paulo, SP Brazil; 10 Postgraduate Program in Public Health, Federal University of Maranhão, São Luís, MA Brazil; 11 Medicine School, Federal University of Goiás, Goiânia, GO Brazil; 12 Department of Nutrition and Dietetics, Josué de Castro Nutrition, Federal University of Rio de Janeiro, Rio de Janeiro, RJ Brazil; 13 Nursing Department, Botucatu Medical School, Julio de Mesquita Filho Paulista State University, Botucatu, SP Brazil; 14 Multidisciplinary Health Institute, Federal University of Bahia, Vitoria da Conquista, BA Brazil; 15 Postgraduate Program in Epidemiology, Federal University of Pelotas, Pelotas, RS Brazil; 16 Public Health Nursing Department, School of Nursing, University of São Paulo, São Paulo, SP Brazil; 17 Department of Obstetrics and Gynecology, School of Medical Sciences, University of Campinas, Campinas, SP Brazil; 18 Department of Health, Feira de Santana State University, Feira de Santana, BA Brazil; 19 Federal University of Rio de Janeiro, Campus Macaé, Macaé, RJ Brazil; 20 Faculty of Nutrition, Federal University of Pelotas, Pelotas, RS Brazil; 21 Postgraduate Program in Public Health, Federal University of Rio Grande, Rio Grande, RS Brazil; 22 Postgraduate Program in Health and Environment, University of Joinville Region, Joinville, SC Brazil; 23 Julio de Mesquita Filho Paulista State University, Botucatu, SP Brazil; 24 Postgraduate Program in Physical Education, Federal University of Pelotas, Pelotas, RS Brazil; 25 Postgraduate Program in Epidemiology, Department of Social Medicine, School of Medicine, Federal University of Rio Grande do Sul, Porto Alegre, RS Brazil; 26 Department of Public Health, Federal University of Maranhão, São Luís, MA Brazil; 27 Health Sciences Center, University of the Joinville Region, Joinville, SC Brazil; 28 Department of Pathology, Medical School of the University of São Paulo, São Paulo, SP Brazil; 29 Department of Epidemiology, Federal University of Recôncavo da Bahia, Santo Antônio de Jesus, BA Brazil

**Keywords:** Epidemiology, Paediatric research

## Abstract

Pooled data analysis in the field of maternal and child nutrition rarely incorporates data from low- and middle-income countries and existing studies lack a description of the methods used to harmonize the data and to assess heterogeneity. We describe the creation of the Brazilian Maternal and Child Nutrition Consortium dataset, from multiple pooled longitudinal studies, having gestational weight gain (GWG) as an example. Investigators of the eligible studies published from 1990 to 2018 were invited to participate. We conducted consistency analysis, identified outliers, and assessed heterogeneity for GWG. Outliers identification considered the longitudinal nature of the data. Heterogeneity was performed adjusting multilevel models. We identified 68 studies and invited 59 for this initiative. Data from 29 studies were received, 21 were retained for analysis, resulting in a final sample of 17,344 women with 72,616 weight measurements. Fewer than 1% of all weight measurements were flagged as outliers. Women with pre-pregnancy obesity had lower values for GWG throughout pregnancy. GWG, birth length and weight were similar across the studies and remarkably similar to a Brazilian nationwide study. Pooled data analyses can increase the potential of addressing important questions regarding maternal and child health, especially in countries where research investment is limited.

## Introduction

The development of pooled analysis with individual patient data has increased worldwide as this practice presents several advantages over the traditional meta-analyses^1^. In 1999, Blettner et al.^2^ highlighted the increasing importance of pooled data analysis. Since then, several initiatives were created, and important scientific evidence has been produced^3–5^.

Open Science and the FAIR (Findable, Accessible, Interoperable, and Reusable) principles dissemination^6^ have promoted strategies for combining resources and data from different studies and become more common in the field of Epidemiology. In low- and middle-income countries (LMIC), however, there are barriers to adhering to the Open Science policy, especially in data sharing. Thus, only a few initiatives using data from LMIC, led by researchers from high-income countries, have been developed recently^7,8^.

In the field of maternal and child nutrition, well-known international collaborations have been established and have led to productive results^9,10^. However, these studies often lack a description of the statistical methods used to harmonize datasets as well as details on how heterogeneity has been assessed. The latter is particularly important given the different origins of the data and techniques applied in data collection^2^.

The Brazilian Maternal and Child Nutrition Consortium (BMCNC) was designed to address these limitations. Its overall goal is to create a large national database on maternal and child nutrition to respond to questions and gaps identified by the Brazilian Ministry of Health and other institutional policy maker agencies. The first project comprises the creation of new gestational weight gain (GWG) recommendations and the development of a new tool to monitor GWG to be used in the Brazilian Unified Health System. In this paper, we describe the creation of the BMCNC dataset derived from multiple pooled and harmonized Brazilian longitudinal studies, describe the characteristics of the study populations included in the consortium, and describe the methods applied for the harmonization of the data in detail, using the example of GWG.

## Results

A total of 11,292 papers were identified in the literature review. Once duplicates were removed, 5,795 papers were screened, and 80 were selected for this study. The search for additional sources revealed 10 new papers/theses that were added to the initial selection. Finally, 90 papers/theses were identified as the result of 68 different studies and projects. Among those, 59 studies were considered eligible to participate in the initiative. From those, 29 PIs did not answer the contact. Among the 30 answers, two were excluded because the study did not fit the inclusion criteria and 28 datasets were requested. We received 18 datasets, and, during this process, 11 new studies were included as suggestions from the contacted PIs. Data from 29 studies were received and initially examined. The profile of the 39 studies not incorporated into the pool revealed that twenty-three (59%) were from the Southeast of Brazil and that 32 out of 39 studies (82%) were conducted after 2000. Maternal age, education, marital status, and pre-pregnancy BMI classification were similar to the observed in the current dataset (data not shown). At the end of the data cleaning process, eight datasets were removed because they did not include gestational age at weight measurements (n = 5) or other essential variables, such as maternal height (n = 3). Thus, twenty-one datasets were retained for further analysis (see Supplementary Fig. S1 online).

**Figure 1:**
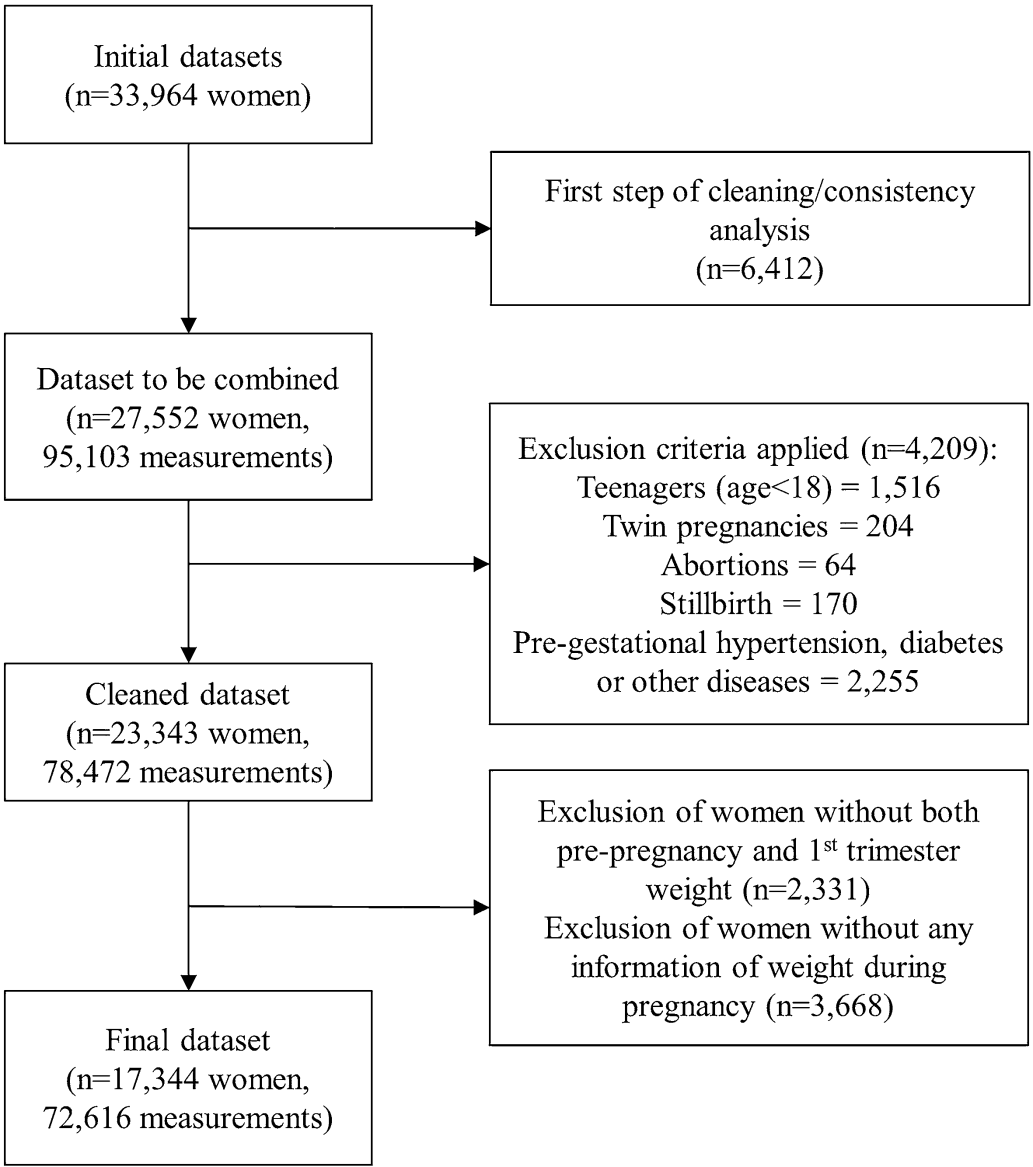
Flowchart for the cleaning steps of the combined dataset.

Pooling the twenty-one datasets produced a cohort of 23,343 women with singleton pregnancy aged 18 years old or older; without pre-pregnancy hypertension, diabetes, HIV, syphilis, thyroid diseases or any other pre-pregnancy disorder that could affect maternal weight; who delivered a liveborn infant. Of these, 2,331 women without data on pre-pregnancy weight or weight measured in the first trimester were excluded because GWG could not be calculated. A total of 3,668 women without any weight measures during pregnancy were also removed, resulting in a final sample of 17,344 women and 72,616 weight measurements in the BMCNC cohort (Fig. 1). These 17,344 women presented remarkably similar characteristics when compared to the 23,343 initially selected (Supplementary table S2 online).

The number of pregnancy weight measures for an individual woman varied from 1 to 19. The methods through which the key variables were collected varied across studies (Table 1). Most studies (71%) collected data from the woman’s pregnancy booklet; maternal height was measured in all of them. Some of the selected studies (24%) collected only self-reported pre-pregnancy weight and a single measure of weight during pregnancy. A complete list of blocks of variables and the number of studies with those data are presented in Supplementary table S3 online.

**Table 1:** Origin of the anthropometric information of the studies included in the Brazilian Maternal and Child Nutrition Consortium.

First author, year	Attributable study name	Original sample size	Maternal height	Maternal pre-pregnancy weight	Maternal weights during pregnancy	Gestational age at weight measurements	Number of pregnancy weight measures	Available outcomes
Schmidt, 2001^34^	EBDG	5,578	Measured	Self-reported	Pregnant booklet/medical records	Calculated according to the standard^b^	14	Birth information
Padilha, 2009^35^	MERJ	1,450	Measured	Self-reported	Pregnant booklet/medical records	Already calculated in the data set	1	Birth information
Nunes, 2010^36^	ECCAGe	716	Measured	Self-reported	Pregnant booklet/medical records	Calculated according to the standard^b^	17	Birth information
Zhang, 2011^37^	EPRG	10,331	Measured	Not used^a^	Interview and medical records	Calculated according to the standard^b^	2	Birth information
Marano, 2012^38^	PQ	1,679	Measured	Self-reported	Pregnant booklet	Calculated according to the standard^b^	12	Birth information, PPCW, PPCL
Santos-Neto, 2012^39^	RMGV	1,035	Measured	Self-reported	Pregnant booklet/medical records	Calculated using LMP date	19	Birth information
Sato, 2012^40^	SP1	228	Measured	Not collected	Pregnant booklet/medical records	Calculated using LMP date	9	Birth information (no sex)
Carvalhaes, 2013^41^	SP2	212	Measured	Self-reported	Pregnant booklet	Calculated according to the standard^b^	13	Birth information
Farias, 2013^42^	RJ	299	Measured	Self-reported	Measured in the visits	Calculated according to the standard^b^	4	Birth information
Figueiredo, 2013^c^	BA1	654	Measured	Self-reported	Pregnant booklet	Already calculated in the data set	1	BW
Santana, 2013^43^	ProcriAr	357	Measured	Self-reported	Measured in the visits	Calculated according to the standard^b^	3	Birth information, PPCW, PPCL
Fernandes, 2014^44^	MEPel	210	Measured	Self-reported	Pregnant booklet/medical records	Already calculated in the data set	2	Birth information
Martinelli, 2014^45^	ES1	742	Measured	Self-reported	Pregnant booklet/medical records	Calculated using LMP date	18	Birth information
Polgliani, 2014^46^	ES2	360	Measured	Self-reported	Pregnant booklet/medical records	Calculated using LMP date	19	Birth information
Carvalhaes, 2015^c^	CLaB	656	Measured	Self-reported	Pregnant booklet/medical records	Already calculated in the data set	2	Birth information, PPCW, PPCL
Magalhaes, 2015^47^	BA2	328	Measured	Self-reported	Measured in the visit	Calculated using LMP date	1	BW
Chagas, 2017^48^	BRISA	1,447	Measured	Self-reported	Measured in the visits	Calculated according to the standard^b^	1	Birth information, PPCW, PPCL
Mastroeni, 2017^49^	PREDI	435	Measured	Self-reported	Measured	Already calculated in the data set	1	Birth information, PPCW
Morais, 2017^50^	SP3	849	Measured	Not collected	Pregnant booklet/medical records	Already calculated in the data set	16	BW
Hallal, 2018^51^	Pelotas	4,329	Measured	Self-reported	Pregnant booklet	Already calculated in the data set	15	Birth information, PPWR, PPCW, PPCL
Morais, 2018^52^	SP4	2,069	Measured	Self-reported	Pregnant booklet	Already calculated in the data set	2	BW, BL

Names of studies are derivated from acronyms and abbreviations from Portuguese: EBDG; Estudo Brasileiro do Diabetes Gestacional (Brazilian Study of Gestational Diabetes); *MERJ* Maternidade-escola, Rio de Janeiro, *ECCAGe* Estudo do Consumo e Comportamento Alimentar na Gestação, *EPRG* Estudos Perinatais de Rio Grande, *PQ* Petrópolis e Queimados, *RMGV* Região Metropolitana da Grande Vitória, *SP1* São Paulo 1, *SP2* São Paulo 2, *RJ* Rio de Janeiro, *BA1* Bahia 1, *ProcriAr* cohort conducted in São Paulo, *MEPel* Maternidade-escola, Pelotas, *ES1* Espírito Santo 1, *ES2* Espírito Santo 2, *CLaB* Coorte de Lactentes de Botucatu, *BA2* Bahia 2, *BRISA* birth cohort in São Luís, Maranhão,*PREDI* PREDIctors of maternal and infant excess body weight—PREDI Study, *SP3* São Paulo 3, *Pelotas* Pelotas 2015 birth cohort, *SP4* São Paulo 4. Birth information (It includes gestational age, *BW* birth weight, *BL* birth length and sex); *PPWR* postpartum weight retention, *PPCW* postpartum child weight, *PPCL* postpartum child length, *LMP* last menstrual period.

^a^self-reported + information copied from the booklet—not used because it was not possible to identify if it was self-reported or not.

^b^Standard: ultrasound estimated age if ultrasound was performed before 24 weeks, LMP date if ultrasound was not available.

^c^Not published study.

Most women were classified as having normal weight before pregnancy (60.1%), delivered term (89.7%) and appropriate for gestational age newborns (74.7%), and had a vaginal delivery (51.8%). In the pooled dataset, 7.4% of the newborns were classified as small for gestational age (SGA), 6.5% as having low birth weight (LBW), and 17.9% as large for gestational age (LGA). Ten percent of women were diagnosed with hypertension during pregnancy and 4.1% with gestational diabetes (Supplementary Table S4 online).

Fewer than 1% of the weight measurements were excluded after being flagged as an outlier by at least one of the adopted methods (0.45% for weight, 0.50% for GWG calculated using first-trimester weight and 0.57% for GWG calculated using self-reported pre-pregnancy weight) (Supplementary Fig. S5 online).

**Figure 2:**
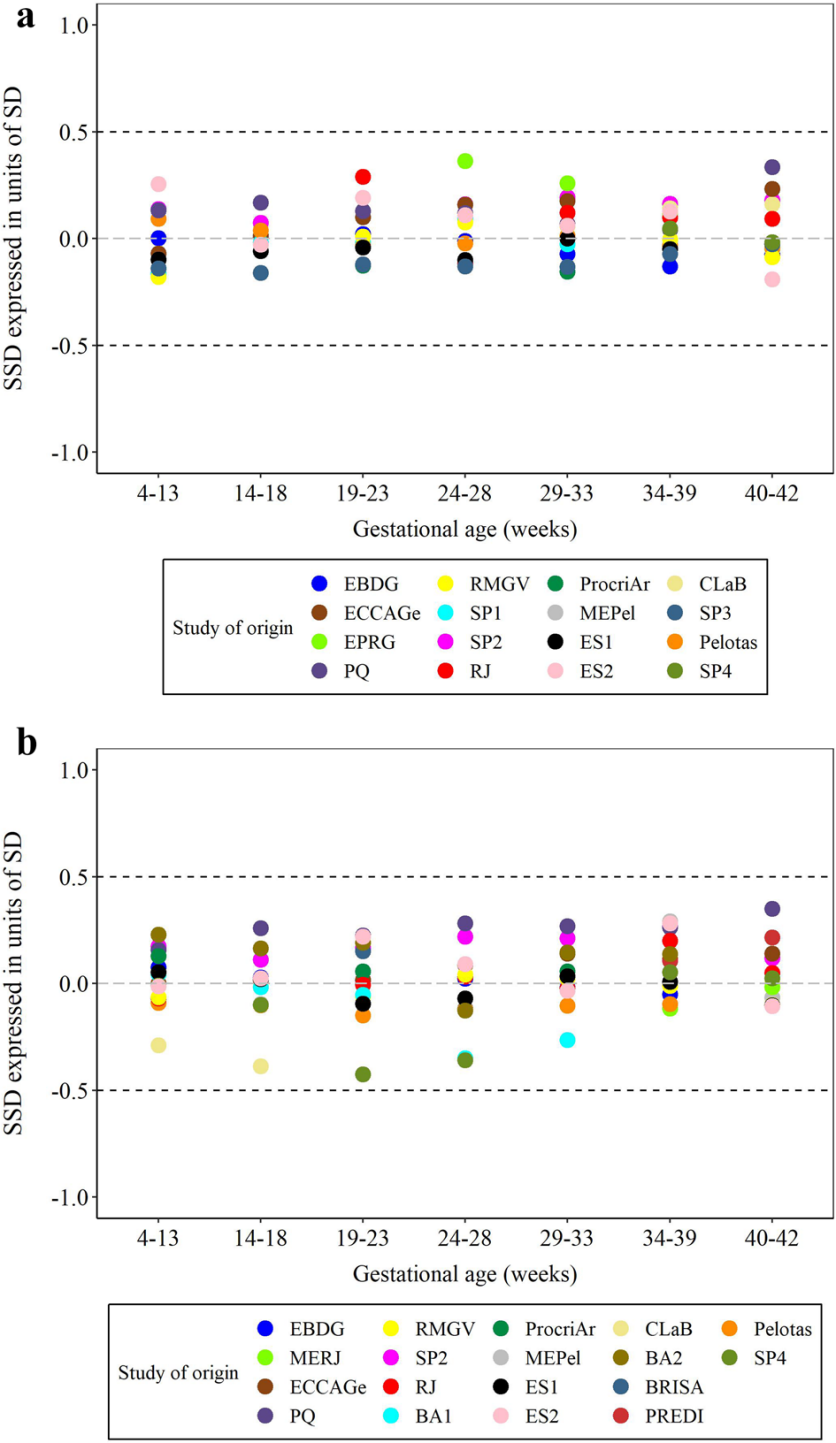
Heterogeneity analysis for the combined datasets: (**a**) Gestational weight gain based on first trimester; (**b**) based on self-reported pre-pregnancy weight. Note: First trimester weight; n = 36,809 measures; Self-reported pre-pregnancy weight: 59,124 measures. Names of studies are derivated from acronyms and abbreviations from Portuguese: EBDG: Estudo Brasileiro do Diabetes Gestacional (Brazilian Study of Gestational Diabetes); *MERJ* Maternidade-escola, Rio de Janeiro, *ECCAGe* Estudo do Consumo e Comportamento Alimentar na Gestação, *EPRG* Estudos Perinatais de Rio Grande, *PQ*: Petrópolis e Queimados, *RMGV* Região Metropolitana da Grande Vitória, *SP1* São Paulo 1, *SP2* São Paulo 2, *RJ* Rio de Janeiro, *BA1* Bahia 1, *ProcriAr* cohort conducted in São Paulo, *MEPel* Maternidade-escola, Pelotas, *ES1* Espírito Santo 1, *ES2* Espírito Santo 2, *ClaB* Coorte de Lactentes de Botucatu, *BA2* Bahia 2, *BRISA* birth cohort in São Luís, Maranhão, *PREDI* PREDIctors of maternal and infant excess body weight—PREDI Study, *SP3* São Paulo 3, *Pelotas* Pelotas 2015 birth cohort, *SP4* São Paulo 4, *SSD* standardized site difference, *SD* standard deviation.

GWG data were highly homogeneous according to the heterogeneity assessment, i.e. ~ 1% of the GWG variance could be explained by the study cohort (Supplementary Table S6 online). When the distribution of GWG across datasets was evaluated according to the GA intervals, all standardized site differences (SSD) values fell within the ± 0.5 SD for both GWG measures, confirming the homogeneity of the data (Fig. 2).

**Figure 3:**
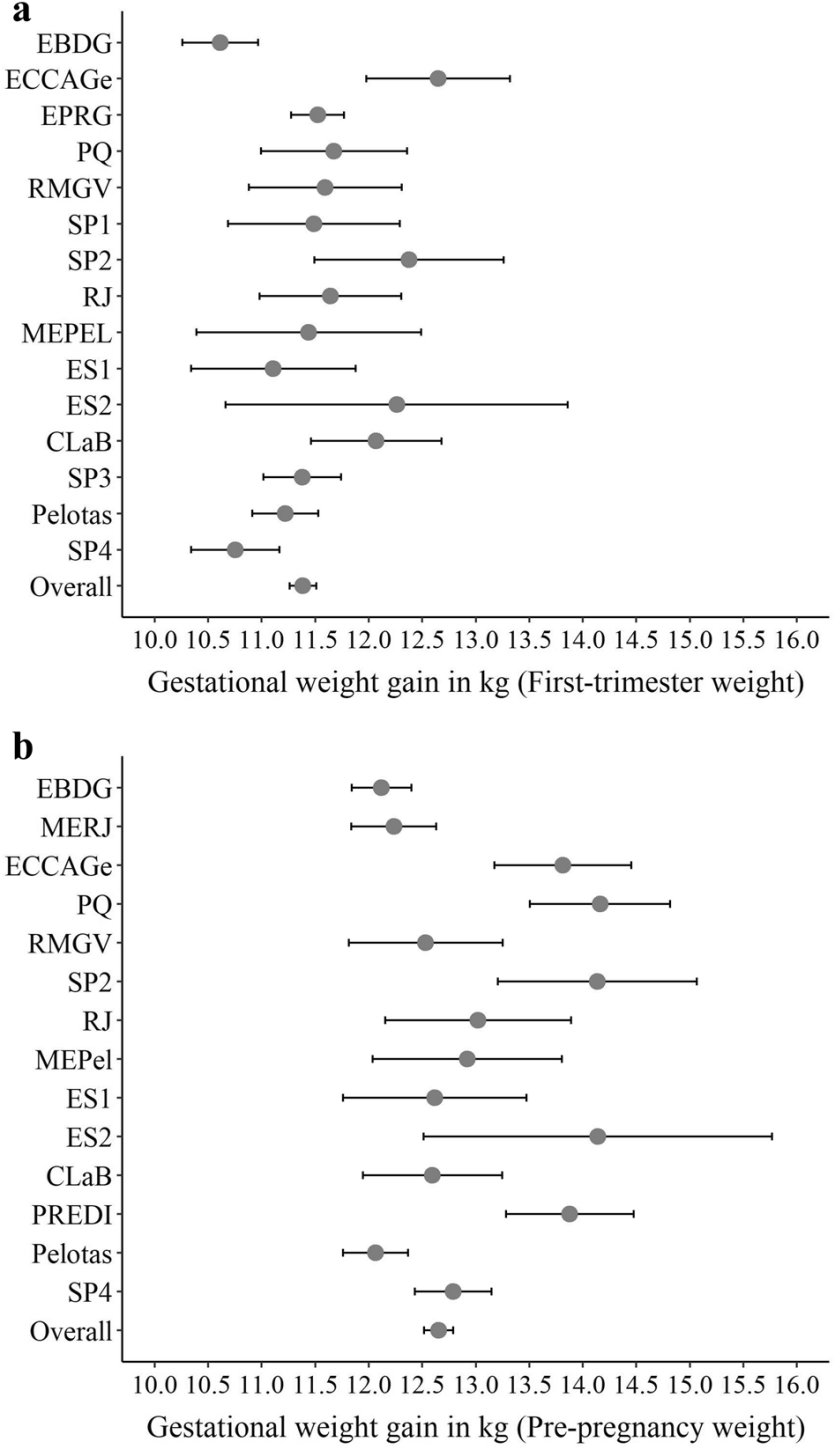
Means and 95% confidence intervals for gestational weight gain calculated using (**a**) first trimester; (**b**) self-reported pre-pregnancy weight. Note: First trimester weight; n = 6,292 women; Self-reported pre-pregnancy weight: 7,426 women. Names of studies are derivated from acronyms and abbreviations from Portuguese: *EBDG* Estudo Brasileiro do Diabetes Gestacional (Brazilian Study of Gestational Diabetes), *MERJ* Maternidade-escola, Rio de Janeiro, *ECCAGe* Estudo do Consumo e Comportamento Alimentar na Gestação, *EPRG* Estudos Perinatais de Rio Grande, *PQ* Petrópolis e Queimados, *RMGV* Região Metropolitana da Grande Vitória, *SP1* São Paulo 1, *SP2* São Paulo 2, *RJ* Rio de Janeiro, *BA1* Bahia 1, *ProcriAr* cohort conducted in São Paulo, *MEPel* Maternidade-escola, Pelotas, *ES1* Espírito Santo 1, *ES2* Espírito Santo 2, *CLaB* Coorte de Lactentes de Botucatu, *BA2* Bahia 2, *BRISA* birth cohort in São Luís, Maranhão, *PREDI* PREDIctors of maternal and infant excess body weight—PREDI Study, *SP3* São Paulo 3, *Pelotas* Pelotas 2015 birth cohort, *SP4* São Paulo 4.

The distribution of total GWG depended on whether self-reported pre-pregnancy weight or weight measured during the first trimester was used. Mean total GWG calculated using first-trimester weight was 11.4 kg (SD: 5.1) and 12.7 (SD: 6.0) for GWG using pre-pregnancy weight (Fig. 3).

**Figure 4:**
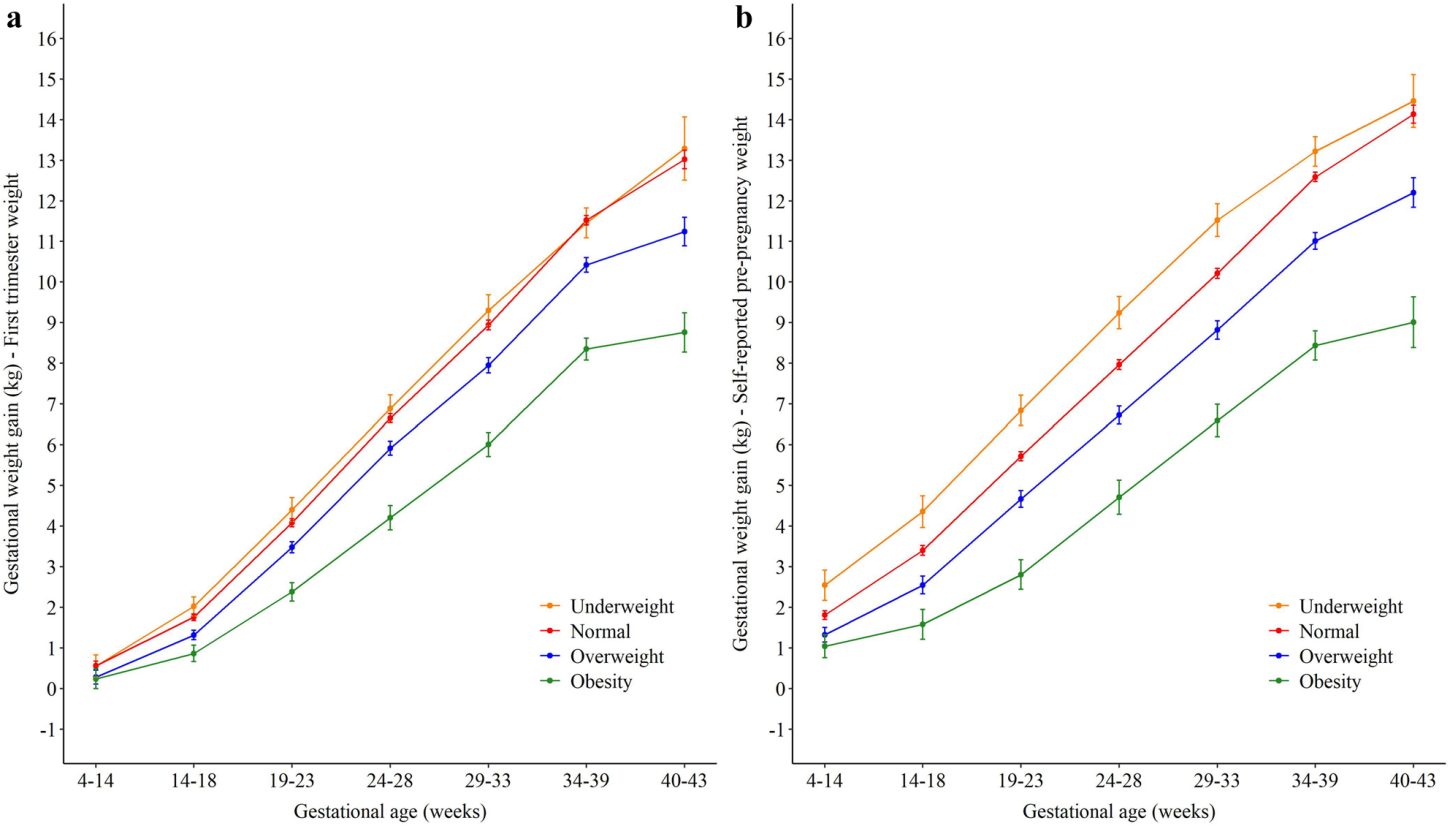
Distribution of weight gain during pregnancy according to (**a**) first trimester; or (**b**) pre-pregnancy body mass index.

The GWG estimates according to body mass index (BMI) category were higher throughout the gestational period when pre-pregnancy weight was used, in comparison to GWG using first trimester weight. Women with obesity had lower GWG at all time points, followed by overweight, normal weight, and underweight women. Using the first-trimester weight, normal and underweight women had similar GWG means at the end of the gestational period. For women with overweight and obesity, the means from 34–39 and 40–42 weeks of gestation had lower increases compared to the other time points, when both GWG measures were evaluated (Fig. 4).

A sensitivity analysis was performed for women with GWG calculated using both pre-pregnancy and first trimester weight (n = 3,526 women). The mean GWG for self-reported weight was 0.5 to 2.3 kg higher than that for measured first trimester weight. The results were also similar when the GWG was evaluated according to BMI categories at the selected time points (Supplementary Table S7 online).

## Discussion

This manuscript presents the creation of the BMCNC. The combined cohort comprises 21 primary studies that collected data between 1990 and 2018 in different regions of Brazil and includes 17,344 pregnant women aged 18 years or older with 72,616 weight measurements. The prevalence of SGA newborns was 7.4% and LGA was 17.9%. Birth characteristics, such as length and weight, did not vary substantially among the studies. GWG differed according to maternal pre-pregnancy BMI and women with obesity presented lower values at all time points, followed by overweight, normal weight and underweight women.

The characteristics of our cohort reflect those of the general Brazilian obstetrical population, especially regarding maternal pre-pregnancy BMI^11^, mean birth weight (3,235 g in this study vs. 3,212 g according to data from the Information System on Live Births, SINASC) and the prevalence of LBW (6.5% in this study and in SINASC)^12^. Our results are also remarkably similar to those observed in Birth in Brazil, a nationwide study conducted in 2011–2012, especially regarding the prevalence of preterm birth (10.3% vs. 11.8% in Birth in Brazil), mode of delivery (51.8% of vaginal delivery vs. 46.4% in Birth in Brazil), and sociodemographic characteristics such as maternal education^13^. Although half of the identified studies were not included in our final cohorts, these similarities reinforce the potential of this data and its representativeness of the country. In addition, when sociodemographic (maternal age, education, marital status) and anthropometric data (pre-pregnancy BMI) of the women from the studies not included in the pool are compared to those from the BMCNC, it is possible to observe that the distributions are quite similar.

There are several advantages of combining studies and conducting a pooled data analysis. One of the key aspects is the increase in sample size, which improves the statistical power of the analyses and thus strengthen the robustness and relevance of the results^14^. Pooling allows better use of the data from individual studies, maximizing the existing resources and, in the case of Brazil, maximizing the public investment made on the individual projects. In addition, it allows researchers to answer questions that the individual studies could not answer themselves. Moreover, pooled data analyses offer an opportunity for collaboration among researchers from different institutions and areas. The creation of consortiums such as ours represents an important data source, especially in countries where the investment in research is limited.

The prevalence of overweight and obesity among pregnant women is increasing worldwide, and most rapidly in middle-income countries, where more than half of women can be classified as overweight^15^. In this pooled cohort, more than 30% of women started pregnancy with overweight or obesity. The trends in GWG according to pre-pregnancy or first trimester BMI were as expected, i.e., women classified with overweight and obesity had lower means values for GWG throughout pregnancy. The total GWG mean (calculated with self-reported pre-pregnancy weight) for women classified with overweight were above the upper limit of the Institute of Medicine (US) recommendations (12.2 kg vs. 11.5 kg recommendation) for overweight women in the US^16^. Ensuring an adequate GWG, especially among heavier women, can contribute to decrease the prevalence of overweight and obesity, since nutritional status during pregnancy is one of the determinants of maternal health^17^.

The prevalence of LGA in this study was 17.9%, which is associated with both pre-pregnancy BMI and the amount of weight gained during pregnancy. Appropriate GWG can help prevent the occurrence of both adverse maternal^18^ and child outcomes, such as the birth of LGA newborns, macrosomia (birth weight > 4,000 g), and obesity during childhood and adolescence^19–21^. Thus, evaluating GWG in developing countries as Brazil, where the prevalence of overweight and obesity is increasing^22^, is especially important and should be part of routine prenatal care.

There is still debate about whether to use self-reported pre-pregnancy weight or first-trimester weight to calculate GWG^23,24^. In this study, when datasets with both types of measures were compared, the differences between them varied from 0.5 to 2.3 kg. Those differences may reflect the amount of weight women are gaining in the first trimester, which was virtually identical to the US Institute of Medicine values of GWG recommended for the first trimester^16^.

The evaluation and consideration of implausible values (outliers) is an issue carefully addressed in this study. Several methods are available in the literature to identify outliers^25^ but dealing with longitudinal measurements can be challenging, as the plausibility of a measurement in relation to that individual’s previous and subsequent measures must also be considered. Two recent approaches were applied^26,27^ and allowed us to flag outliers in the women’s trajectories and values that were discrepant from the general distribution. We considered the combination of methods efficient because only those measurements that really seemed implausible were flagged as outliers. The exclusion of a low percentage of measurements flagged as outliers had minimal impact on the distribution of GWG and produced more plausible longitudinal data.

The homogeneity of the GWG data provided reassurance that this harmonized cohort can be used to perform robust analyses and respond to many other objectives of the BMCNC. The initiative to combine datasets from different studies is not new for GWG. Santos et al.^9^ have harmonized several European cohorts with GWG data. Although these authors used a combination of different datasets, few details were provided about how they assessed heterogeneity. In the current paper, all steps for the creation of a pooled dataset were reported, so that they can be used in future studies. The code used in the harmonization process is available upon request from the corresponding author.

This cohort has the potential to address a broad range of maternal and child health research questions. The large number of women, with repeated measures of weight during pregnancy, and, for a sub-cohort, with a postpartum follow-up of both mothers and their children, are some of the strengths of the combined cohort described here. The detection of outliers adopted in the study, which included approaches incorporating the longitudinal characteristics of the data is a strength of this work as is the evaluation of GWG heterogeneity across the datasets, which is usually not performed in studies of this nature. The similarity between birth outcomes and maternal characteristics with other Brazilian data reinforces the generalizability of this cohort.

Unfortunately, only half of the eligible studies could be incorporated into the combined cohort. In a few cases, the principal investigator (PI) was no longer active, and it was not possible to recover the dataset. The main reason that studies could not be included was a lack of response to the invitation to participate. This was unfortunate because some of these studies were carried out in underexplored regions of the country and would have been welcomed to fill spaces left somewhat unattended. The fact that each study used a different procedure to collect some of the key variables for the main purpose of this analysis, such as gestational age, is a constraint when evaluating the pooled data, but we tried to address this problem through careful harmonization of the variables.

## Methods

### Identification of studies

We conducted a literature review including papers published from January 1990 to December 2018 to identify studies eligible for the BMCNC initiative. Search strategies were created for PubMed/Medline, Web of Science, Scopus, LILACS, and *Scielo* (a Latin-American Scientific Library) to identify Brazilian studies that have measured weight or weight gain during pregnancy. Search strategies included the terms: pregnancy/gestation (and variations); Brazil; epidemiologic studies; cohort/longitudinal/prospective/observational (and variations). We also searched for cross-sectional, case–control studies, and clinical trials since they could have GWG information to be used in the current study. Additional searches were performed in the Lattes Platform (a Brazilian database with information on science, technology, and innovation), to identify ongoing or unpublished projects. To be included in the BMCNC, the studies must have been approved by a research ethics committee; have an observational study design and have been conducted in Brazil after 1990, have pre-pregnancy or first-trimester body mass index (BMI) and weight during pregnancy, have been conducted with adult women (≥ 18 years old), free of infectious diseases, and have a sample size of at least 100 women.

The identified publications were downloaded to a library in EndNote, where duplicates were identified and removed. A reviewer selected the studies based on the titles and abstracts of the manuscripts. Full texts were consulted whenever necessary. A second reviewer verified 10% of the discarded studies to ensure that no eligible study was eliminated by mistake. This procedure did not uncover any new results. A team of four reviewers checked all the selected publications to confirm that they met the inclusion criteria for the BMCNC. To perform this step, the following information was extracted from the manuscripts (when available): location and period of the study, sample size, number of pregnancy visits/weight measures, maternal and child outcomes, other variables of interest (such as sociodemographic characteristics), origin of the anthropometric measures (self-reported, measured, medical records), availability of pre-pregnancy weight data and eligibility criteria.

After eligibility confirmation, the study PI was identified and invited by e-mail to participate in the initiative. In the same e-mail, a standardized form was used to request additional information about the studies. After the replies were received, a list of predetermined variables from the study dataset was requested. Once the dataset and data dictionary were received and checked, the distribution of the variables was evaluated to identify implausible values or discrepancies. The PIs were contacted whenever there were questions or problems with the data received.

### Creation of a pooled dataset

To construct a pooled dataset, the first step of the cleaning process comprised an analysis of the consistency of the data, which was performed cross-sectionally and longitudinally. In this step, for each dataset, essential variables (such as dates of visits and weights) were checked for chronological order, statistical distribution, and missing data. Gestational age (at visits and birth) was standardized and calculated according to the ultrasound performed before 24 weeks of gestation or the date of the last menstrual period if the former was unavailable. In some datasets, it was not possible to calculate the gestational age according to the specified criteria, because the dates were not available (only the age already calculated).

Additionally, a dictionary of variables based on all studies was created to standardize the format and units of measure in the different datasets (such as weight in kilograms, gestational age in days). These datasets were then combined, and the frequency of all variables was examined to evaluate distribution similarities and differences.

### Creation of variables

Following the harmonization of the datasets, derived variables were created, which ensured that this process was consistent across the studies. Cumulative GWG was calculated in two ways: first, by the difference between the weight measured in any visit and the first measure of weight during the first trimester; and, second, by the difference between the weight measured in any visit and the self-reported pre-pregnancy weight. Total GWG was calculated using the same procedures and only women with weight measured within 14 days of delivery were considered for this variable.

BMI (kg/m^2^) was calculated dividing the weight (first trimester or self-reported pre-pregnancy) in kg by the measured height in meters squared. Nutritional status based on BMI was classified according to the World Health Organization (WHO) cutoffs^28^ as underweight (< 18.5 kg/m^2^), normal (≥ 18.5 and < 25.0 kg/m^2^), overweight (≥ 25.0 and < 30.0 kg/m^2^) and obese (≥ 30.0 kg/m^2^).

Birth weight (g) was categorized as SGA (< 10th percentile) or LGA (> 90th percentile) for gestational age by using the sex-specific INTERGROWTH-21st neonatal charts^29^. In addition, the prevalence of low birth weight (LBW, < 2,500 g) was determined. Z scores for length at birth were also calculated according to INTERGROWTH-21st charts^29^. Gestational age at birth was classified as preterm (< 37 weeks) and term (≥ 37 weeks)^30^. Information on mode of delivery, hypertension, and diabetes during pregnancy were used as binary variables. The way that information was collected varied by study and was either reported by women or measured in the study.

### Statistical analyses

A detailed evaluation of outliers was conducted for the weight and GWG variables. Three procedures were implemented. The conditional method proposed by Yang and Hutcheon^26^ was initially used to identify outliers in the distribution considering the longitudinal nature of the data. This approach flags outliers that are four standard deviations (SD) above or below the estimated individual’s conditional mean, using a random-effects model. Moreover, unconditional means were also used to flag observations that were ± 4 SD from those values.

The third approach used to identify outlying values was a modified version of the methodology proposed by Shi et al.^27^, which flags as outliers the visits where the jackknife (or studentized) residuals are out of the ± 4 range after each women’s weight or GWG is regressed as a function of gestational age in women-specific models. The original approach was modified to flag jackknife residuals out of this range in relation to weight and GWG distribution adjusted for gestational age considering the whole dataset. The combination of methods was necessary to identify women who only had a single measure of weight at very extreme values of the distribution (and would not be flagged as outliers by using the conditional means method). All approaches identified visits where weight or GWG measurements were implausible. These procedures allowed us to remove only the specific data point considered to be an outlier. We removed the measurements flagged as outliers if they represented a percentage below 2% of the total data, given the impossibility of verifying the values in the original data sources.

To check if the harmonization process was appropriate and assess the heterogeneity of GWG distribution across datasets, multilevel models of GWG that included gestational age and study cohort (adjusted or not by BMI) were fitted. The percentage of the GWG variance explained by the original cohort was then determined. Additionally, SSD were compared across datasets by calculating the *z* scores for the means of GWG in gestational age groups (4–13, 14–18, 19–23, 24–28, 29–33, 34–39, 40–42 gestational weeks) in relation to the pooled means and SDs for each age group, in a similar approach to that adopted by WHO^31^ and INTERGROWTH-21st^32^. The dataset was considered homogeneous if values of SSD were between − 0.5 and + 0.5, a cut-off also used by WHO in the Multicentre Growth Reference Study^31^. According to Cohen^33^, differences of 0.5 SD units are considered medium, while differences of 0.2 SD units are small and 0.8 are large. For this analysis, each dataset contributed to specific time points, but not necessarily the same ones.

When assessing heterogeneity, we excluded all observations from a particular study in the gestational age groupings where the sample size for that study included fewer than 30 women after the cleaning procedure was implemented. This decision was made because smaller datasets could contribute too highly for heterogeneity as a result of the small sample size rather than true biological heterogeneity. This restriction was also applied when evaluating the total GWG (datasets with n < 30 were not included in the graphs).

After evaluating outliers and the heterogeneity of GWG data, the variable distributions were evaluated using means, SDs, and 95% confidence intervals (continuous variables) and absolute and relative frequencies (categorical variables). As a result of the large sample size, statistical tests to compare the distribution of the variables according to the datasets were not performed. We also compared the distribution (means, SDs/absolute, relative frequencies) of sociodemographic variables and pregnancy outcomes between the 23,343 (dataset without removing missing data in weight) and the 17,344 women selected for this study. Analyses were conducted in both Stata (version 15) and R (version 3.5).

### Ethics approval

The Research Ethics Committee of the Rio de Janeiro Federal University Maternity Teaching Hospital approved this study (Protocol Number: 85914318.2.0000.5275) and all analyses were conducted with deidentified data to preserve the confidentiality of individuals’ information. Additionally, all incorporated studies were individually approved by their own institutional research ethics committees, informed consent was obtained from the participants of each study and they were conducted in accordance with the principles of the Declaration of Helsinki.

## Supplementary information

Supplementary Information.

## Data Availability

The data that support the findings of this study are available from the Brazilian Maternal and Child Nutrition Consortium, but restrictions apply to the availability of these data, which were used under license for the current study, and so are not publicly available yet. Data are however available from the authors upon reasonable request and with permission of all members of the Consortium.
