# Supplementary material for: Brazilian Maternal and Child Nutrition Consortium: establishment, data harmonization and basic characteristics
Source: Sci Rep. 2020 Sep 10;10:14869. doi: 10.1038/s41598-020-71612-8 (PMC7483530; doi:10.1038/s41598-020-71612-8)
Supplement: Supplementary file 1 — Supplementary Information. [file 41598_2020_71612_MOESM1_ESM.pdf]

Supplementary information

Brazilian Maternal and Child Nutrition Consortium: Establishment, data harmonization and basic characteristics

Authors: Thaís Rangel Bousquet Carrilho, Dayana Rodrigues Farias, Mônica Araújo Batalha, Nathalia Cristina Freitas Costa, Kathleen M. Rasmussen, Michael E. Reichenheim, Eric O. Ohuma, Jennifer A. Hutcheon, Gilberto Kac, Brazilian Maternal and Child Nutrition Consortium.

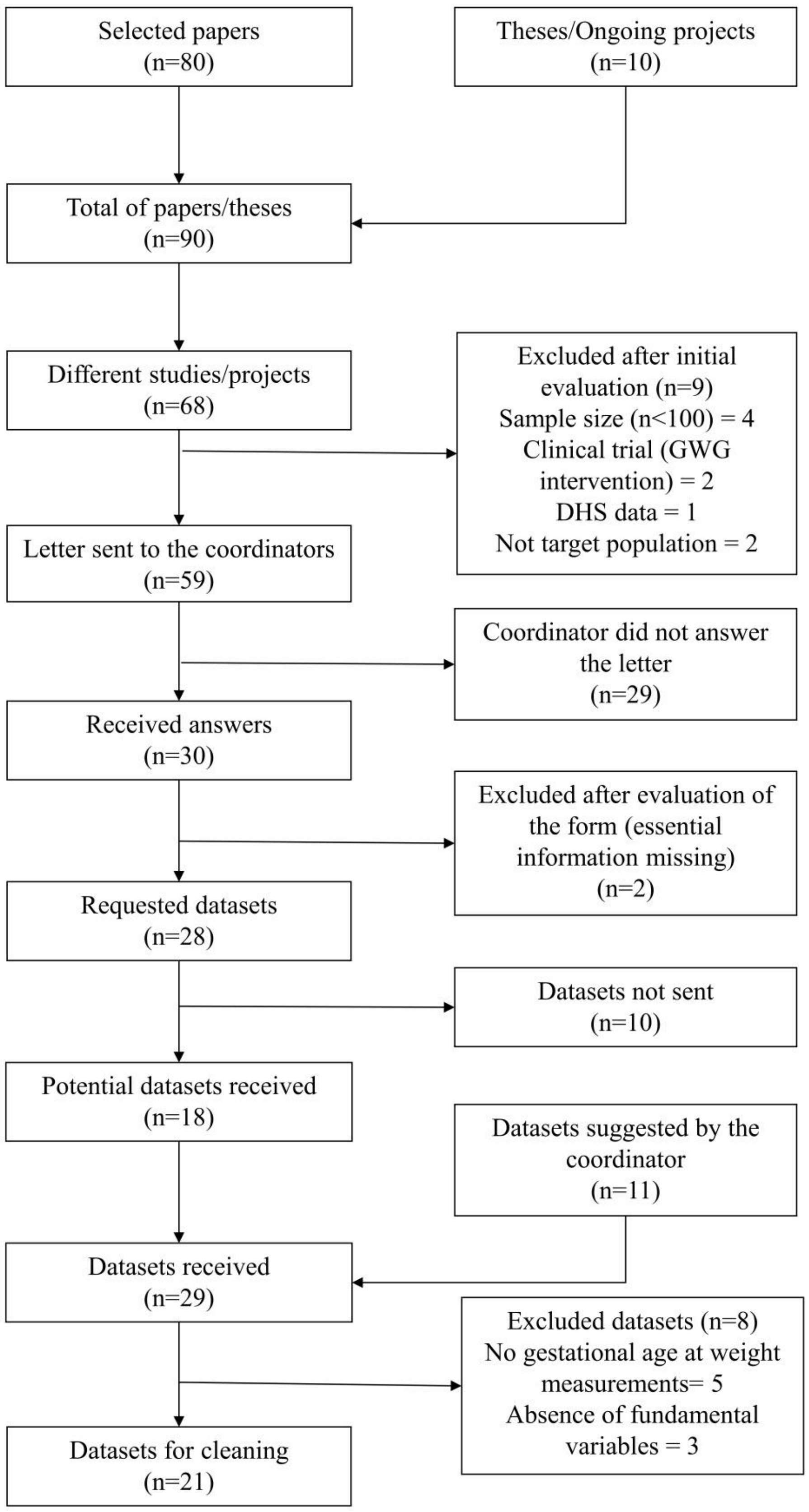

Supplementary figure S1. Flowchart for the acquisition of datasets. Note: GWG: gestational weight gain; DHS: Demographic and Health Surveys.

Supplementary table S2. Comparison of women before and after cleaning first-trimester and self-reported pre-pregnancy weight, data from the Brazilian Maternal and Child Nutrition Consortium.

Continuous variables	Before cleaning* (23,343 women)	After cleaning* (17,344 women)
Maternal Age (n)	22,199	17,326
Mean (SD)	26.8 (5.8)	26.9 (5.8)
Min; Max	18; 49	18; 47
Gestational age at first visit (days) (n)	19,675	17,344
Mean (SD)	117.1 (62.0)	113.3 (62.9)
Min; Max	30; 299	30; 299
Gestational age at birth (days) (n)	20,805	16,671
Mean (SD)	272.4 (15.1)	272.8 (14.2)
Min; Max	168; 301	169; 301
Birth weight (g) n	21,435	16,683
Mean (SD)	3,229.1 (521.0)	3,235.4 (513.0)
Min; Max	500; 5,650	500; 5,250
Birth length (cm) n	18,043	14,034
Mean (SD)	48.6 (2.6)	48.7 (2.6)
Min; Max	26; 61	26; 61
Categorical variables	n (%)	n (%)
First-trimester nutritional status (BMI, kg/m^2)	8,812	8,812
Underweight (<18.5)	360 (4.1)	360 (4.1)
Normal (18.5 – 24.9)	4,488 (50.9)	4,488 (50.9)
Overweight (25 – 29.9)	2,587 (29.4)	2,587 (29.4)
Obese (> 30)	1,377 (15.6)	1,377 (15.6)
Pre-pregnancy nutritional status (BMI, kg/m^2)	15,233	13,272
Underweight (<18.5)	975 (6.4)	825 (6.2)
Normal (18.5 – 24.9)	9,210 (60.4)	7,981 (60.1)
Overweight (25 – 29.9)	3,440 (22.6)	3,032 (22.9)
Obese (> 30)	1,608 (10.6)	1,434 (10.8)
Maternal education (schooling years)	21,119	16,475
< 4	2,418 (11.4)	1,775 (10.8)
4 - 8	5,888 (27.9)	4,447 (27.0)
9 - 11	9,922 (47.0)	7,895 (47.9)

≥ 12	2,891 (13.7)	2,358 (14.3)
Marital status	15,350	12,397
Lives with a partner	13,081 (85.2)	10,585 (85.4)
Does not live with a partner	2,269 (14.8)	1,812 (14.6)
Mode of delivery	21,247	16,522
Normal	11,028 (51.9)	8,552 (51.8)
Cesarean	10,219 (48.1)	7,970 (48.2)
Hypertension (during pregnancy)	22,429	16,886
Yes	2,273 (10.1)	1,716 (10.2)
No	20,156 (89.9)	15,170 (89.8)
Diabetes (during pregnancy)	21,556	16,837
Yes	861 (4.0)	699 (4.1)
No	20,695 (96.0)	16,138 (95.9)

Note: *Cleaning refers to the removal of missing data in weight during pregnancy and pre-pregnancy weight or weight measured in the first trimester. 1. Variation in the number of individuals in each category is due to missing. 2. SD: standard deviation; Min: minimum; Max: maximum.

Supplementary table S3. Variables available in the studies included in the Brazilian Maternal and Child Nutrition Consortium.

Life-cycle period	Blocks of variables	Total of studies
Pre-pregnancy	Sociodemographic/economic	21
	Obstetric history	20
	Lifestyle	11
	Maternal health	8
	Maternal mental health	2
	Food consumption	1
	Maternal anthropometry	8
	Use of medicine/nutritional supplements	3
Pregnancy	Lifestyle	15
	Maternal health	15
	Maternal mental health	4
	Food consumption	11
	Use of medicine/nutritional supplements	10
	Biochemistry (blood samples)	8
	Blood pressure	8
	Maternal anthropometry	16
	Ultrasonography data	5
Birth	Birth information	17
	Maternal health	5
	Child's health	9
	Maternal anthropometry	4
	Child's anthropometry	13
Post-partum	Lifestyle	4
	Maternal health	6
	Maternal mental health	4
	Maternal food consumption	1
	Maternal biochemistry (blood samples)	0
	Maternal blood pressure	2
	Maternal anthropometry	5
	Child's health	6
	Child's food consumption/breastfeeding	8
	Use of medicine/nutritional supplements (mother/child)	2
	Child's biochemistry (blood samples)	1
	Child's anthropometry	6

Supplementary table S4. General description of the studies included in the Brazilian Maternal and Child Nutrition Consortium. (Part 1)

	**Combined n (%)**	**EBDG n (%)**	**MERJ n (%)**	**ECCAGe n (%)**	**EPRG n (%)**	**PQ n (%)**
1st trimester nutritional status (BMI, kg/m^2)	8,812	1,345	Not measured	249	2,275	299
Underweight (<18.5)	360 (4.1)	67 (5.0)		7 (2.8)	94 (4.1)	20 (6.7)
Normal (18.5 – 24.9)	4,488 (50.9)	870 (64.7)		139 (55.8)	1,081 (47.5)	162 (54.2)
Overweight (25 – 29.9)	2,587 (29.4)	341 (25.3)		66 (26.5)	672 (29.6)	72 (24.1)
Obese (≥ 30)	1,377 (15.6)	67 (5.0)		37 (14.9)	428 (18.8)	45 (15.0)
Pre-pregnancy nutritional status (BMI, kg/m^2)	13,272	3,888	712	474	Not measured	532
Underweight (<18.5)	825 (6.2)	268 (6.9)	28 (3.9)	18 (3.8)		43 (8.1)
Normal (18.5 – 24.9)	7,981 (60.1)	2,726 (70.1)	396 (55.6)	291 (61.4)		338 (63.5)
Overweight (25 – 29.9)	3,032 (22.9)	727 (18.7)	191 (26.8)	114 (24.0)		96 (18.1)
Obese (≥ 30)	1,434 (10.8)	167 (4.3)	97 (13.6)	51 (10.8)		55 (10.3)
Age (n)	17,326	3,888	711	476	2,490	580
Mean (SD)	26.9 (5.8)	27.3 (5.2)	28.9 (5.7)	25.7 (5.8)	26.5 (5.9)	25.8 (5.9)
Min; Max	18; 47	20; 46	19; 45	18; 42	18; 45	18; 45
Gestational age at birth (weeks) n	16,671	3,442	712	474	2,476	577
Mean (SD)	39.0 (2.0)	39.1 (2.1)	39.1 (1.6)	39.0 (2.1)	38.9 (2.4)	39.0 (2.2)
Min; Max	24.1; 43	25.7; 43	27.3; 42.7	24.9; 43	24.1; 43	26; 42.9

Classification of gestational age	16,671	3,442	712	474	2,476	577
Preterm birth (< 37 weeks)	1,710 (10.3)	398 (11.6)	41 (5.8)	61 (12.9)	348 (14.0)	72 (12.5)
Term birth (≥ 37 weeks)	14,961 (89.7)	3,044 (88.4)	671 (94.2)	413 (87.1)	2,128 (86.0)	505 (87.5)
Birth weight (g) n	16,683	3,387	712	475	2,489	575
Mean (SD)	3,235.3 (513.0)	3,214.5 (526.9)	3,276.7 (487.3)	3,248.7 (520.0)	3,209.2 (558.9)	3,210.8 (504.0)
Min; Max	500; 5,250	840; 5,250	915; 4,970	1,250; 5,050	515; 5,230	500; 5,220
n (base for SGA/LGA)	16,523	3,375	712	473	2,474	574
SGA	1,216 (7.4)	338 (10.0)	36 (5.1)	37 (7.8)	183 (7.4)	41 (7.1)
LGA	2,966 (17.9)	579 (17.2)	122 (17.1)	100 (21.1)	470 (19.0)	100 (17.4)
LBW (<2500g)	1,082 (6.5)	252 (7.4)	32 (4.5)	36 (7.6)	197 (7.9)	43 (7.5)
Birth length (cm) n	14,034	2,971	701	426	2,430	541
Mean (SD)	48.7 (2.6)	49.1 (2.6)	48.8 (2.4)	48.6 (2.6)	48.0 (2.8)	48.5 (2.8)
Min; Max	26.0; 61.0	36.0; 61.0	34.50; 61.0	32.0; 55.5	28.0; 56.0	26.0; 57.0
Birth length (z-score) n	13,919	2,961	701	425	2,419	540
Mean (SD)	0.1 (1.3)	0.3 (1.4)	0.1 (1.2)	0.1 (1.4)	-0.2 (1.4)	0.1 (1.4)
Min; Max	-6.5; 6.7	-5.8; 5.2	-5.6; 5.2	-6.0; 3.9	-6.4; 6.7	-5.1; 5.8
Mode of delivery	16,522	3,393	621	467	2,490	580
Vaginal	8,552 (51.8)	2,197 (64.8)	305 (49.1)	291 (62.3)	1,108 (44.5)	311 (53.6)

Cesarean	7,970 (48.2)	1,196 (35.2)	316 (50.9)	176 (37.7)	1,382 (55.5)	269 (46.4)
Hypertension during pregnancy	16,886	3,888	712	476	2,490	580
Yes	1,716 (10.2)	157 (4.0)	49 (6.9)	19 (4.0)	433 (17.4)	104 (17.9)
No	15,170 (89.8)	3,721 (96.0)	663 (93.1)	457 (96.0)	2,057 (82.6)	476 (82.1)
Diabetes during pregnancy	16,837	3,887	712	476	2,490	579
Yes	699 (4.1)	18 (0.5)	220 (30.9)	3 (0.6)	96 (3.9)	14 (2.4)
No	16,138 (95.9)	3,869 (99.5)	492 (69.1)	473 (99.4)	2,394 (96.1)	565 (97.6)

Note: Names of studies are derivated from acronyms and abbreviations from Portuguese: EBDG; Estudo Brasileiro do Diabetes Gestacional (Brazilian Study of Gestational Diabetes); MERJ: Maternidade-escola, Rio de Janeiro; ECCAGe: Estudo do Consumo e Comportamento Alimentar na Gestação; EPRG: Estudos Perinatais de Rio Grande; PQ: Petrópolis e Queimados; RMGV: Região Metropolitana da Grande Vitória; SP1: São Paulo 1; SP2: São Paulo 2; RJ: Rio de Janeiro; BA1: Bahia 1; ProcriAr: cohort conducted in São Paulo; MEPel: Maternidade-escola, Pelotas; ES1: Espírito Santo 1; ES2: Espírito Santo 2; CLaB: Coorte de Lactentes de Botucatu; BA2: Bahia 2; BRISA: birth cohort in São Luís, Maranhão; PREDI: PREDIctors of maternal and infant excess body weight - PREDI Study; SP3: São Paulo 3; Pelotas: Pelotas 2015 birth cohort; SP4: São Paulo 4. BMI: Body Mass Index; SD: standard deviation; Min: minimum; Max: maximum; SGA: small for gestational age; LGA: large for gestational age; LBW: low birth weight. Variation in the number of individuals in each category is due to missing.

Supplementary table S4. General description of the studies included in the Brazilian Maternal and Child Nutrition Consortium. (Part 2)

	**RMGV n (%)**	**SP1 n (%)**	**SP2 n (%)**	**RJ n (%)**	**BA1 n (%)**	**ProcriAr n (%)**
1st trimester nutritional status (BMI, kg/m^2)	160	162	153	225	129	306
Underweight (<18.5)	13 (8.2)	6 (3.7)	6 (3.9)	7 (3.1)	4 (3.1)	7 (2.3)
Normal (18.5 – 24.9)	73 (45.6)	106 (65.4)	84 (54.9)	123 (54.7)	67 (51.9)	136 (44.4)
Overweight (25 – 29.9)	45 (28.1)	42 (25.9)	43 (28.1)	68 (30.2)	40 (31.0)	102 (33.3)
Obese (≥ 30)	29 (18.1)	8 (5.0)	20 (13.1)	27 (12.0)	18 (14.0)	61 (20.0)
Pre-pregnancy nutritional status (BMI, kg/m^2)	297	Not measured	194	234	530	315
Underweight (<18.5)	28 (9.4)		14 (7.2)	12 (5.1)	40 (7.6)	10 (3.2)
Normal (18.5 – 24.9)	168 (56.9)		117 (60.3)	133 (56.9)	300 (56.6)	168 (53.3)
Overweight (25 – 29.9)	57 (19.2)		43 (22.2)	60 (25.6)	124 (23.4)	84 (26.7)
Obese (≥ 30)	43 (14.5)		20 (10.3)	29 (12.4)	66 (12.4)	53 (16.8)
Age (n)	458	162	194	241	536	325
Mean (SD)	25.4 (5.7)	25.4 (5.2)	26.1 (5.5)	26.5 (5.3)	26.8 (6.1)	26.9 (5.8)
Min; Max	18; 42	18; 40	18; 43	20; 40	18; 42	18; 42
Gestational age at birth (weeks) n	437	158	185	225	478	321
Mean (SD)	38.9 (2.9)	40.0 (1.3)	39.3 (1.9)	38.6 (2.1)	39.1 (1.6)	39.2 (1.5)
Min; Max	24.1; 43	29.4; 42.1	26.3; 42.1	28; 43	30; 43	32; 41.9

Classification of gestational age	437	158	185	225	478	321
Preterm birth (< 37 weeks)	76 (17.4)	1 (0.6)	12 (6.5)	29 (87.1)	16 (3.4)	24 (7.5)
Term birth (≥ 37 weeks)	361 (82.6)	157 (99.4)	173 (93.5)	196 (12.9)	462 (96.6)	297 (92.5)
Birth weight (g) n	447	162	194	225	485	325
Mean (SD)	3,231.3 (564.5)	3,277.7 (520.0)	3,207.7 (514.6)	3,246.2 (557.8)	3,281.3 (498.3)	3,238.3 (460.7)
Min; Max	750; 5,050	2,180; 4,530	660; 4,630	1320; 4,635	980; 5,150	1,540; 4,630
n (base for SGA/LGA)	425	158	185	224	467	321
SGA	42 (9.9)	19 (12.0)	11 (6.0)	10 (4.5)	35 (7.5)	27 (8.4)
LGA	96 (22.6)	20 (12.2)	19 (10.3)	46 (20.5)	88 (18.8)	49 (15.3)
LBW (<2500g)	37 (8.3)	4 (2.5)	12 (6.2)	16 (7.1)	18 (3.7)	16 (4.9)
Birth length (cm) n	437			222		290
Mean (SD)	48.7 (2.5)	Not measured	Not measured	49.5 (3.2)	Not measured	48.4 (2.3)
Min; Max	34.0; 57.0			33.0; 57.0		41.0; 54.0
Birth length (z-score) n	415			221		286
Mean (SD)	0.2 (1.4)	Not measured	Not measured	0.7 (1. 6)	Not measured	-0.2 (1.3)
Min; Max	-3.6; 5.4			-5. 8; 5.8		-4.4; 3.9
Mode of delivery	455	127	194	222	486	295
Vaginal	271 (59.6)	85 (66.9)	133 (68.6)	124 (55.9)	200 (41.)	153 (51.9)
Cesarean	184 (40.4)	42 (33.1)	61 (31.4)	98 (44.1)	286 (58.9)	142 (48.1)

Hypertension during pregnancy	333	Not measured	194	211	535	192
Yes	28 (8.4)		0	2 (0.1)	21 (3.9)	13 (6.8)
No	305 (91.6)		194 (100)	209 (99.0)	514 (96.1)	179 (93.2)
Diabetes during pregnancy	298		194	208	535	192
Yes	10 (3.4)	Not measured	0	13 (6.3)	6 (1.1)	5 (2.6)
No	288 (96.6)		194 (100)	195 (93.7)	529 (98.9)	187 (97.4)

Note: Names of studies are derivated from acronyms and abbreviations from Portuguese: EBDG; Estudo Brasileiro do Diabetes Gestacional (Brazilian Study of Gestational Diabetes); MERJ: Maternidade-escola, Rio de Janeiro; ECCAGe: Estudo do Consumo e Comportamento Alimentar na Gestação; EPRG: Estudos Perinatais de Rio Grande; PQ: Petrópolis e Queimados; RMGV: Região Metropolitana da Grande Vitória; SP1: São Paulo 1; SP2: São Paulo 2; RJ: Rio de Janeiro; BA1: Bahia 1; ProcriAr: cohort conducted in São Paulo; MEPel: Maternidade-escola, Pelotas; ES1: Espírito Santo 1; ES2: Espírito Santo 2; CLaB: Coorte de Lactentes de Botucatu; BA2: Bahia 2; BRISA: birth cohort in São Luís, Maranhão; PREDI: PREDIctors of maternal and infant excess body weight - PREDI Study; SP3: São Paulo 3; Pelotas: Pelotas 2015 birth cohort; SP4: São Paulo 4. BMI: Body Mass Index; SD: standard deviation; Min: minimum; Max: maximum; SGA: small for gestational age; LGA: large for gestational age; LBW: low birth weight. Variation in the number of individuals in each category is due to missing.

Supplementary table S4. General description of the studies included in the Brazilian Maternal and Child Nutrition Consortium. (Part 3)

	**MEPel**<br>**n (%)**	**ES1**<br>**n (%)**	**ES2**<br>**n (%)**	**CLaB**<br>**n (%)**	**BA2**<br>**n (%)**
1st trimester nutritional status (BMI, kg/m^2)	101	238	38	362	37
Underweight (<18.5)	3 (3.0)	19 (8.0)	1 (2.6)	6 (1.7)	3 (8.1)
Normal (18.5 – 24.9)	57 (56.4)	121 (50.8)	23 (60.5)	156 (43.1)	19 (51.4)
Overweight (25 – 29.9)	24 (23.8)	64 (26.9)	11 (29.0)	116 (32.0)	10 (27.0)
Obese (≥ 30)	17 (16.8)	34 (14.3)	3 (7.9)	84 (23.2)	5 (13.5)
Pre-pregnancy nutritional status (BMI, kg/m^2)	200	343	72	392	271
Underweight (<18.5)	15 (7.5)	34 (9.9)	1 (1.4)	13 (3.3)	24 (8.9)
Normal (18.5 – 24.9)	109 (54.5)	184 (53.6)	49 (68.1)	192 (49.0)	163 (60.1)
Overweight (25 – 29.9)	45 (22.5)	77 (22.5)	15 (20.8)	104 (26.5)	63 (23.3)
Obese (≥ 30)	31 (15.5)	48 (14.0)	7 (9.7)	83 (21.2)	21 (7.7)
Age (n)	206	496	103	488	272
Mean (SD)	25.8 (5.7)	25.5 (5.4)	25.4 (5.8)	28.0 (6.2)	25.4 (5. 7)
Min; Max	18; 41	18; 42	18; 38	18; 42	18; 42
Gestational age at birth (weeks) n	206	476	93	467	256
Mean (SD)	39.4 (1.2)	39.3 (1.9)	38.6 (2.8)	38.9 (1.5)	38.9 (1.8)
Min; Max	37; 43	26.4; 43	26.7; 42.9	30; 42	30; 43
Classification of gestational age	206	476	93	467	256

Preterm birth (< 37 weeks)	0	44 (9.2)	14 (15.1)	18 (3.9)	22 (8.6)
Term birth (≥ 37 weeks)	206 (100)	432 (90.8)	79 (84.9)	449 (96.1)	234 (91.4)
Birth weight (g) n	206	495	103	486	256
Mean (SD)	3,299.4 (479.3)	3,313.3 (454.8)	3,194.7 (438.4)	3,239.2 (456.5)	3,204.4 (514.8)
Min; Max	2,060; 4,750	800; 5,080	1,600; 4,390	1,520; 4,725	650; 4,780
n (base for SGA/LGA)	205	474	93	467	253
SGA	13 (6.3)	35 (7.4)	11 (11.8)	25 (5.4)	19 (7.5)
LGA	31 (15.2)	97 (20.5)	15 (16.1)	77 (16.5)	42 (16.6)
LBW (<2500g)	9 (4.4)	19 (3.8)	4 (3.9)	24 (4.9)	15 (5.9)
Birth length (cm) n		487	102	477	
Mean (SD)	Not measured	49.7 (3.1)	48.1 (2.4)	48.8 (2.3)	Not measured
Min; Max		31.0; 57.0	40.0; 53.0	32.5; 57.0	
Birth length (z-score) n		467	92	458	
Mean (SD)	Not measured	0.6 (1.5)	-0.2 (1.6)	0.2 (1.1)	Not measured
Min; Max		-6.0; 4.6	-4.5; 5.3	-6.3; 4.1	
Mode of delivery	206	496	102	489	255
Vaginal	110 (53.4)	209 (42.1)	64 (62.8)	240 (49.1)	171 (67.1)
Cesarean	96 (46.6)	287 (57.9)	38 (37.2)	249 (50.9)	84 (32.9)

Hypertension during pregnancy	206	496	101	491	272
Yes	0	53 (10.7)	2 (2.0)	4 (0.8)	7 (2.6)
No	206 (100)	443 (89.3)	99 (98.0)	487 (99.2)	265 (97.4)
Diabetes during pregnancy	206	496	95	491	272
Yes	0	2 (0.4)	4 (4.2)	2 (0.4)	0
No	206 (100)	494 (99.6)	91 (95.8)	489 (99.6)	272 (100)

Note: Names of studies are derivated from acronyms and abbreviations from Portuguese: EBDG; Estudo Brasileiro do Diabetes Gestacional (Brazilian Study of Gestational Diabetes); MERJ: Maternidade-escola, Rio de Janeiro; ECCAGe: Estudo do Consumo e Comportamento Alimentar na Gestação; EPRG: Estudos Perinatais de Rio Grande; PQ: Petrópolis e Queimados; RMGV: Região Metropolitana da Grande Vitória; SP1: São Paulo 1; SP2: São Paulo 2; RJ: Rio de Janeiro; BA1: Bahia 1; ProcriAr: cohort conducted in São Paulo; MEPel: Maternidade-escola, Pelotas; ES1: Espírito Santo 1; ES2: Espírito Santo 2; CLaB: Coorte de Lactentes de Botucatu; BA2: Bahia 2; BRISA: birth cohort in São Luís, Maranhão; PREDI: PREDIctors of maternal and infant excess body weight - PREDI Study; SP3: São Paulo 3; Pelotas: Pelotas 2015 birth cohort; SP4: São Paulo 4. BMI: Body Mass Index; SD: standard deviation; Min: minimum; Max: maximum; SGA: small for gestational age; LGA: large for gestational age; LBW: low birth weight. Variation in the number of individuals in each category is due to missing.

Supplementary table S4. General description of the studies included in the Brazilian Maternal and Child Nutrition Consortium. (Part 4)

	**BRISA n (%)**	**PREDI n (%)**	**SP3 n (%)**	**Pelotas n (%)**	**SP4 n (%)**
1st trimester nutritional status (BMI, kg/m^2)	3	Not measured	589	1,396	745
Underweight (<18.5)	0		32 (5.4)	37 (2.6)	28 (3.8)
Normal (18.5 – 24.9)	1 (33.3)		283 (48.0)	615 (44.2)	372 (49.9)
Overweight (25 – 29.9)	2 (66.7)		180 (30.6)	453 (32.4)	236 (31.7)
Obese (≥ 30)	0		94 (16.0)	291 (20.8)	109 (14.6)
Pre-pregnancy nutritional status (BMI, kg/m^2)	1,001	425	Not measured	2,296	1,096
Underweight (<18.5)	99 (9. 9)	20 (4.7)		91 (4.0)	67 (6.1)
Normal (18.5 – 24.9)	670 (66.9)	252 (59.3)		1,116 (48.6)	608 (55.5)
Overweight (25 – 29.9)	185 (18.5)	100 (23.5)		665 (28.9)	282 (25.7)
Obese (≥ 30)	47 (4.7)	53 (12.5)		424 (18.5)	139 (12.7)
Age (n)	1,140	425	651	2,366	1,118
Mean (SD)	26.1 (5.1)	25. 9 (6.1)	26.1 (5.7)	27.9 (6.3)	27.7 (5.9)
Min; Max	18; 43	18; 42	18; 42	18; 47	18; 44
Gestational age at birth (weeks) n	1,139	425	657	2,365	1,102
Mean (SD)	39.4 (1.7)	39.1 (1.1)	37.8 (2.7)	38.8 (1.9)	39.1 (1.5)
Min; Max	24.9; 42,9	37; 42	24.3; 41.6	25; 42.9	28.9; 42.3
Classification of gestational age	1,139	425	657	2,365	1,102

Preterm birth (< 37 weeks)	68 (6.0)	0	148 (22.5)	264 (11.2)	54 (4.9)
Term birth (≥ 37 weeks)	1,071 (94.0)	425 (100)	509 (77.5)	2,101 (88.8)	1,048 (95.1)
Birth weight (g) n	1,134	425	645	2,362	1,095
Mean (SD)	3,261.7 (485.6)	3,417.5 (446.9)	3,202.7 (555.7)	3,216.8 (508.6)	3,239.6 (449.5)
Min; Max	930; 4,780	2,100; 5,220	625; 5,010	645; 5,065	1,350; 4,645
n (base for SGA/LGA)	1,133	425	644	2,361	1,080
SGA	86 (7.6)	10 (2.4)	33 (5.1)	134 (5.7)	71 (6.6)
LGA	160 (14.1)	103 (24.2)	183 (28.4)	419 (17.8)	150 (13.9)
LBW (<2500g)	63 (5.6)	3 (0.7)	61 (9.5)	157 (6.6)	64 (5.9)
Birth length (cm) n	1,103	425		2,357	1,065
Mean (SD)	49.2 (2.4)	48.6 (1.8)	Not measured	48.4 (2.4)	48.6 (2.2)
Min; Max	33.0; 57.0	40.0; 54.5		28.9; 56.7	29.0; 55.0
Birth length (z-score) n	1,102	425		2,356	1,051
Mean (SD)	0.3 (1.2)	-0.1 (1.1)	Not measured	0.1 (1.1)	-0.1 (1.1)
Min; Max	-6.1; 4.8	-4.4; 3.1		-6.1; 4.7	-6.5; 3.5
Mode of delivery	1,138	425	627	2,366	1,088
Vaginal	576 (50.6)	287 (67.5)	368 (58.7)	917 (38.8)	432 (39.7)
Cesarean	562 (49.4)	138 (32.5)	259 (41.3)	1449 (61.2)	656 (60.3)

Hypertension during pregnancy	1,139	425	662	2,365	1,118
Yes	161 (14.1)	22 (5.2)	13 (2.0)	544 (23.0)	84 (7.5)
No	978 (85.9)	403 (94.8)	649 (98.0)	1,821 (77.0)	1,034 (92.5)
Diabetes during pregnancy	,1140	425	662	2,366	1,113
Yes	22 (1.9)	29 (6.8)	5 (0.8)	192 (8.1)	58 (5.2)
No	1,118 (98.1)	396 (93.2)	657 (99.2)	2,174 (91.9)	1055 (94.8)

Note: Names of studies are derivated from acronyms and abbreviations from Portuguese: EBDG; Estudo Brasileiro do Diabetes Gestacional (Brazilian Study of Gestational Diabetes); MERJ: Maternidade-escola, Rio de Janeiro; ECCAGe: Estudo do Consumo e Comportamento Alimentar na Gestação; EPRG: Estudos Perinatais de Rio Grande; PQ: Petrópolis e Queimados; RMGV: Região Metropolitana da Grande Vitória; SP1: São Paulo 1; SP2: São Paulo 2; RJ: Rio de Janeiro; BA1: Bahia 1; ProcriAr: cohort conducted in São Paulo; MEPel: Maternidade-escola, Pelotas; ES1: Espírito Santo 1; ES2: Espírito Santo 2; CLaB: Coorte de Lactentes de Botucatu; BA2: Bahia 2; BRISA: birth cohort in São Luís, Maranhão; PREDI: PREDIctors of maternal and infant excess body weight - PREDI Study; SP3: São Paulo 3; Pelotas: Pelotas 2015 birth cohort; SP4: São Paulo 4. BMI: Body Mass Index; SD: standard deviation; Min: minimum; Max: maximum; SGA: small for gestational age; LGA: large for gestational age; LBW: low birth weight. Variation in the number of individuals in each category is due to missing.

a

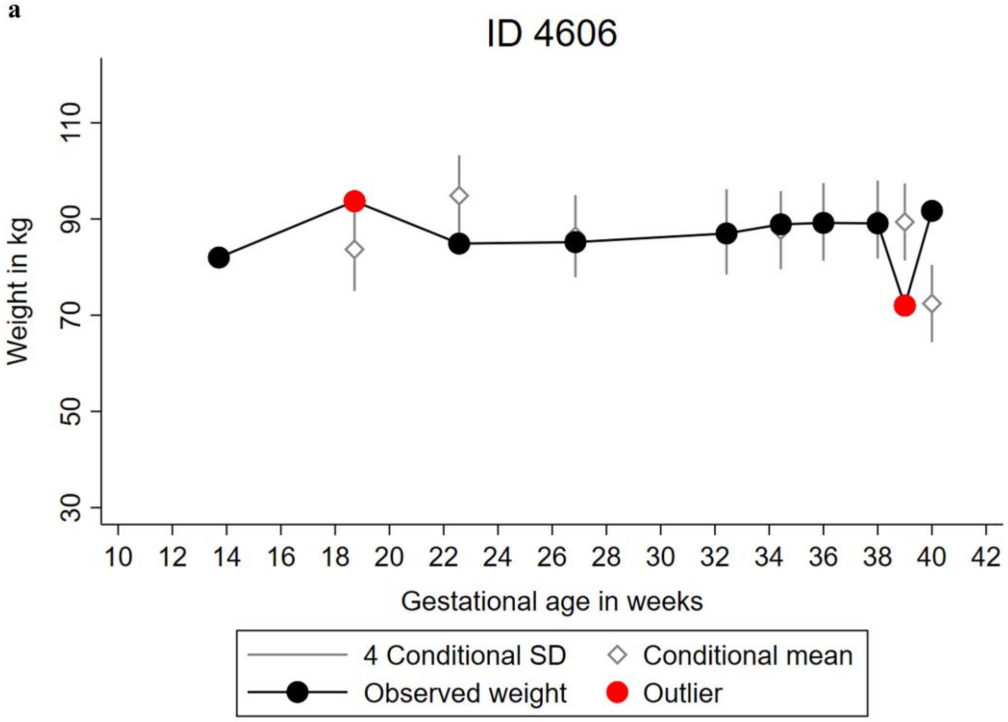

b

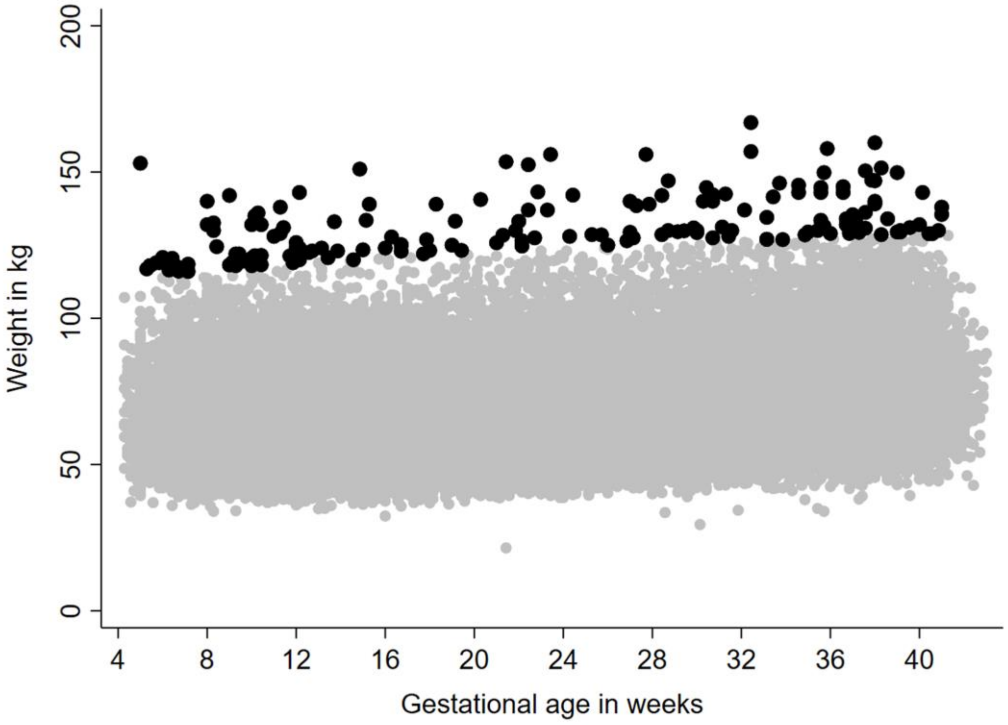

Supplementary figure S5. Examples of outliers for maternal weight during pregnancy. a. Conditional mean approach using 4 standard deviation (SD); b. Jackknife residuals using 4 as cutoff.

Supplementary table S6. Alternative approach for heterogeneity assessment of the studies included in Brazilian Maternal and Child Nutrition Consortium.

	**GWG (First trimester weight) Absolute value (%)**	**GWG (pre-pregnancy weight) Absolute value (%)**
Crude variance due to the original cohort	0.1 (0.5)	0.2 (0.8)
Crude variance due to id	13.0	21.6
Crude Residual variance	3.4	3.8
Adjusted models		
Adjusted variance due to the original cohort*	0.1 (0.8)	0.2 (0.8)
Adjusted variance due to id*	11.9	20.4
Adjusted Residual variance*	3.4	3.8
Adjusted variance due to the original cohort **	0.1 (0.8)	0.2 (0.9)
Adjusted variance due to id**	11.8	20.2
Adjusted Residual variance**	3.4	3.8

* Adjusted for nutritional status based on Body Mass Index (BMI) (calculated using 1st trimester weight or self-reported pre-pregnancy weight).
** Adjusted for pre-pregnancy BMI (continuous, calculated using 1st trimester weight or self-reported pre-pregnancy weight).
Note: GWG: Gestational weight gain.

Supplementary table S7. Comparative analysis between total gestational weight gain calculated using first trimester weight or self-reported pre-pregnancy weight (n=3,526 women).

Study name	GWG First-trimester weight (kg)		GWG self-reported pre-pregnancy weight (kg)		Difference between means (kg)
	Mean	95% CI	Mean	95% CI	
EBDG	10.6	10.3 - 11.0	12.5	12.1 - 13.0	1.93
ECCAGe	12.7	12.0 -13.4	14.3	13.5 - 15.1	1.61
PQ	11.7	11.0 - 12.4	13.9	13.0 - 14.9	2.27
RMGV	11.7	10.8 - 12.5	12.6	11.7 - 13.6	0.96
SP2	12.4	11.5 – 13.3	14.4	13.3 – 15.4	1.98
RJ	11.7	11.0 - 12.4	13.2	12.3 - 14.1	1.49
MEPel	11.4	10.4 - 12.5	12.8	11.56 - 14.1	1.38
ES1	11.1	10.3 - 11.9	12.9	11.9 - 13.9	1.78
CLAB	12.2	11.5 - 12.9	12.8	12.0 - 13.5	0.56
PELOTAS	11.2	10.9 - 11.6	12.3	11.9 - 12.6	1.01
SP4	11.4	11.0 - 11.7	13.0	12.6 - 13.4	1.60
Overall	11.4	11.3 - 11.6	12.9	12.7 - 13.1	1.46

Note: Names of studies are derivated from acronyms and abbreviations from Portuguese: EBDG; Estudo Brasileiro do Diabetes Gestacional (Brazilian Study of Gestational Diabetes); ECCAGe: Estudo do Consumo e Comportamento Alimentar na Gestação; PQ: Petrópolis e Queimados; RMGV: Região Metropolitana da Grande Vitória; SP2: São Paulo 2; RJ: Rio de Janeiro; MEPel: Maternidade-escola, Pelotas; ES1: Espírito Santo 1; CLaB: Coorte de Lactentes de Botucatu; Pelotas: Pelotas 2015 birth cohort; SP4: São Paulo 4. GWG: gestational weight gain; CI: confidence interval.
